# Editorial: Wellbeing and quality of life in elite sports: Towards evidence-based approaches for psychological health promotion and proactive support

**DOI:** 10.3389/fpsyg.2022.1071183

**Published:** 2022-11-21

**Authors:** Carolina Lundqvist, Nick Galli, Abbe Brady

**Affiliations:** ^1^Department of Behavioral Sciences and Learning, Linköping University, Linköping, Sweden; ^2^Department of Health, Athletics Research Center, Medicine and Caring Sciences, Linköping University, Linköping, Sweden; ^3^Department of Health and Kinesiology, University of Utah, Salt Lake City, UT, United States; ^4^Department of Psychology and Pedagogic Science, St Mary's University, London, United Kingdom

**Keywords:** elite athlete, elite coach, mental health, wellbeing, Olympic sports

## Introduction

Wellbeing and mental health are hot topics in sports with an increasing number of studies published each year and significant media attention. During the Tokyo 2020 Olympic Games (OG), Simone Biles, known as the greatest gymnast of all times, sent an important message to the world of sports: Elite athletes are humans sometimes facing crushing pressures. Under high media attention, Biles interrupted her participation in the women's gymnastics team final and canceled several individual OG finals to protect her mental health and ensure her physical safety. Biles tweeted: “I'm more than my accomplishments and gymnastics which I never truly believed before” (@simone_Biles, 2021, July 29).

Contemporary research reports several interrelated risk-factors for athlete mental health within elite sports-systems (Purcell et al., 2019). Risk-factors include, for example, organizational stressors, stigma related to mental health problems, normalization of unhealthy behaviors, injuries, career dissatisfaction, harassment and abuse, and toxic sports leadership or other dysfunctional relationships (e.g., Mountjoy et al., 2016; Reardon et al., 2019; Kuettel and Larsen, 2020). Increased commercialization and professionalization, together with stakeholders and media, place further demands and obligations on athletes (Timpka et al., 2008).

Most sport-specific risk factors for mental health in elite sports environments are known to be modifiable, and the collection of studies published in recent years points to the need for researchers to study support systems for mental health promotion and prevention. Such efforts should focus on proactive support increasing athlete wellbeing and resiliency in response to both expected and naturally occurring stressors in the environment and support the development of targeted prevention and treatments when mental wellbeing or mental disorders are suspected (e.g., Purcell et al., 2019; Lundqvist et al., 2022).

This Research Topic includes an important collection of articles spanning authors from several continents with contributions targeting mental health from a variety of conceptual and theoretical perspectives. Several articles have a direct application for researchers and practitioners working with mental health support in various elite athlete populations and cultures.

This Research Topic includes:

- Conceptual considerations of mental health research
- Wellbeing promotion, mental health prevention and treatment
- Empirical studies targeting critical processes or career phases for athlete mental health.

## Conceptual considerations of mental health research

Pointing to the complexity of separating symptoms associated with normal or expected mood variations in relation to sports performance and symptoms that signal mental illness or mental disorders, Lundqvist and Andersson provide an overview of theoretical perspectives commonly adopted in mental health research in sports. Theoretical perspectives on mental health, with strengths and limitations discussed, include wellbeing as a target construct, holistic models, single continuum and stage models, Keyes' dual-continuum model and the psychiatric/biomedical view.

Theoretical perspectives targeting career terminations are also discussed by Wendling and Sagas. The authors present an integrative framework of self-reformulation and discuss developmental changes and psychosocial processes essential for elite athletes' identity reformation during the transition out of elite sports when discovering a new meaningful identity in life after sports.

## Wellbeing promotion, mental health prevention, and treatment

In this Research Topic five articles focus on support systems or frameworks for mental health support and provide excellent examples of approaches from four continents: Asia, Europe, North America, and Oceania.

In an article written by Purcell et al. contemporary research is summarized in an evidence-informed framework with a whole system approach. Promotion of healthy environments are encouraged together with recommendations on how mental wellbeing among both athletes and staff involved in elite sports environments can be obtained.

Van Slingerland and Durand-Bush present an evaluation of practitioners and service-users view of acceptability and appropriateness of a sport-centered and collaborative mental health service delivery model implemented within the Canadian Center for Mental Health and Sports.

The current state of Japanese athletes' wellbeing and level of knowledge about the topic, together with perceptions of support services is presented by Noguchi et al. in a pilot study. The results indicate needs of education programs, guidelines, detection system and information accessibility related to wellbeing and mental health in the Japanese elite sports system.

Hoare et al. provide a community case study proposing a Mental Fitness Model based on the PERMA model of wellbeing for implementation in Australian youth high performance settings. The model is suggested to support wellbeing and promote mental health needs of young high-level athletes.

Finally, Ekelund et al. raise concerns that evidence on the effectiveness of psychotherapeutic interventions on athletes are lacking, and discuss definitions and procedures used to determine prevalence rates of mental health problems and disorders.

## Empirical studies of critical processes or career phases

Four empirical papers are included in this Research Topic. Bennie et al. interviewed 18 Australian Olympic athletes about their post-Olympic experiences after the Rio 2016 Olympic Games. Factors that positively influenced athlete wellbeing included that performance appraisal met expectations, planning of return to work or studies, and readily available support.

Wu et al. investigated associations between motivational processes, psychological distress, and burnout in a sample of 685 winter sports athletes. Results revealed a task-oriented motivational climate to be positively related to basic needs, and autonomous and controlled motivation to show a negative association with symptoms of psychological distress and burnout.

Attachment relationships to significant others were investigated by Davis et al. in different samples of athletes representing various sports and skill levels. The results supported the importance of secure attachment relationships with parents and coaches related to thriving among athletes.

Finally, Tubić et al. investigated the prevalence of psychological distress among elite and recreational sambo athletes. Results revealed elite sambo athletes to report significantly lower scores on self-rated depression, anxiety, stress, and general distress than their recreational counterpart.

Mental health is a priority in elite sports. We are therefore proud to conclude that the 11 articles in this Research Topic collectively capture several novel and future-oriented perspectives contributing to the increasing knowledge base of mental health promotion and proactive support efforts within elite sports environments.

## Author contributions

CL wrote the first draft of the Editorial. All authors read and revised the draft and approved the final version of the Editorial.

## Conflict of interest

The authors declare that the research was conducted in the absence of any commercial or financial relationships that could be construed as a potential conflict of interest.

## Publisher's note

All claims expressed in this article are solely those of the authors and do not necessarily represent those of their affiliated organizations, or those of the publisher, the editors and the reviewers. Any product that may be evaluated in this article, or claim that may be made by its manufacturer, is not guaranteed or endorsed by the publisher.
